# The Potential of Molecular Indicators of Plant Virus Infection: Are Plants Able to Tell Us They Are Infected?

**DOI:** 10.3390/plants11020188

**Published:** 2022-01-11

**Authors:** Gardette R. Valmonte-Cortes, Sonia T. Lilly, Michael N. Pearson, Colleen M. Higgins, Robin M. MacDiarmid

**Affiliations:** 1 School of Science, AUT City Campus, Auckland University of Technology, Auckland 1142, New Zealand; colleen.higgins@aut.ac.nz; 2 The New Zealand Institute for Plant & Food Research Limited, 120 Mt Albert Road, Auckland 1025, New Zealand; soniatlilly@gmail.com (S.T.L.); robin.macdiarmid@plantandfood.co.nz (R.M.M.); 3 School of Biological Sciences, The University of Auckland, Thomas Building, 3a Symonds Street, Auckland 1010, New Zealand; m.pearson@auckland.ac.nz

**Keywords:** plant virus, detection, sRNA, molecular markers, CPK, *SGS3*, host response

## Abstract

To our knowledge, there are no reports that demonstrate the use of host molecular markers for the purpose of detecting generic plant virus infection. Two approaches involving molecular indicators of virus infection in the model plant *Arabidopsis thaliana* were examined: the accumulation of small RNAs (sRNAs) using a microfluidics-based method (Bioanalyzer); and the transcript accumulation of virus-response related host plant genes, suppressor of gene silencing 3 (*AtSGS3*) and calcium-dependent protein kinase 3 (*AtCPK3*) by reverse transcriptase-quantitative PCR (RT-qPCR). The microfluidics approach using sRNA chips has previously demonstrated good linearity and good reproducibility, both within and between chips. Good limits of detection have been demonstrated from two-fold 10-point serial dilution regression to 0.1 ng of RNA. The ratio of small RNA (sRNA) to ribosomal RNA (rRNA), as a proportion of averaged mock-inoculation, correlated with known virus infection to a high degree of certainty. *AtSGS3* transcript decreased between 14- and 28-days post inoculation (dpi) for all viruses investigated, while *AtCPK3* transcript increased between 14 and 28 dpi for all viruses. A combination of these two molecular approaches may be useful for assessment of virus-infection of samples without the need for diagnosis of specific virus infection.

## 1. Introduction

Virus infections in plants pose a major threat to agricultural, environmental, and economic security globally [1,2]. Strict monitoring of viruses and other pathogens that may arrive with plant materials is implemented as part of border biosecurity by several/many countries [3,4]. In-field monitoring of viruses (and other pathogens) is also becoming increasingly important [5,6]. However, available detection tools are limited, particularly with regards to plant virus detection. Current available assays are limited to symptom observation, visualisation of virus particles, and serological and molecular tests to detect virus particles or nucleic acids [7]. Symptom observation may be inaccurate and insufficient to differentiate viral infections from other types of pathogen infection or abiotic stress [8]. Symptom observation and visualisation of virions require inoculation of plant tissue samples into indicator plants, which is time consuming and dependent on the viability of inoculum. Serological and molecular tests target specific viruses and require previous knowledge of viruses likely to be present and/or virus sequence to develop appropriate nucleic acid-based or protein-based assays for each virus or group of viruses [8,9]. High throughput sequencing is promising as a method of virus detection but is currently deemed too expensive for routine diagnostics [5,7,8,10]. The use of nano sensing technologies is recently being explored, however current technology is limited to the detection of specific and known viruses [6].

Rather than detecting specific viruses, we hypothesise that molecular markers involved in host plant cellular responses to viruses may be more advantageous as they do not require viral sequence information to identify infected plant or plant material. Recently, indirect detection of viral infections through host response-related molecular markers has been explored in several human and animal studies, but not yet in plants. In humans, the use of host signatures to detect and/or discriminate viral infections, such as protein biomarkers, gene transcripts, and other molecular signals have been recently reported [11,12,13,14,15].

Plants respond to viruses through a network of genetic and intracellular pathways, including RNA silencing or interference (RNAi) [16,17,18]. During plant virus infection, replicative viral double-stranded RNAs (dsRNAs) trigger host plant RNAi to cleave dsRNAs into virus-derived small interfering RNAs (siRNAs) that are 20–25 nucleotides (nt) in size [19,20]. Virus-derived siRNAs target cognate viral siRNAs for cleavage/degradation as part of the host’s defence against viruses [21,22]. Given the antiviral nature of these virus-derived siRNAs, it is not surprising that an increase in the amount of accumulating small RNA (sRNA) or of specific sRNAs have been reported in association with plant virus infections [18,21,22,23,24,25,26,27].

Identification and profiling of small RNAs using deep-sequencing methods have also been explored as an approach to diagnose and control plant virus infections in recent studies [20,27,28,29,30,31,32]. Detection of plant virus-infection by deep sequencing of sRNA was considered comparable to virus detection based on next-generation sequencing [29,33]. Deep-sequencing of sRNA was also reported to be more sensitive than virus-specific reverse-transcriptase quantitative PCR (RT-qPCR) assays in detecting Potato virus A (PVA) and Y (PVY) [29]. Aside from deep sequencing, microfluidics or lab-on-chip technologies such as the Agilent Bioanalyzer 2100 platform coupled with Agilent Pico or Nano RNA kits (Agilent Technologies, Santa Clara, CA, USA) might be suitable for the quantification of sRNA accumulation. This platform is increasingly being used to rapidly assess RNA quality and quantity for qPCR experiments [34] and thus may be promising as a quick tool to quantify sRNA. This includes known and unknown sRNAs, endogenous and exogenous, that is, total low molecular weight RNA (LMW-RNA).

In addition to sRNA, specific plant host genes may also be useful as molecular markers for virus infection. Recent studies have utilised microarray, transcriptomics, proteomics and metabolomics approaches to identify host genes and molecular signatures that differentially respond to virus infections [35,36,37,38,39,40,41,42]. In *Arabidopsis thaliana*, suppressor of gene silencing 3 (*AtSGS3*) has been reported to have a role in the RNA pathway to amplify the dsRNA signal [43] and involved in defence responses against several plant viruses [44]. The orthologue of *SGS3* in *Nicotiana benthamiana*, was reported to cooperate with RNA-dependent RNA polymerase 6 (RDR6) in fighting against virus infection [44,45]. Moreover, *SGS3* mRNA levels have been reported to show positive correlation with viral RNA levels in *SGS3*-silenced and *SGS3*-overexpressing leaves of *N. benthamiana* infected with PVA [46]. Hence, *SGS3* may be a good candidate as a molecular indicator of virus infection. Another gene in *A. thaliana*, calcium-dependent protein kinase 3 (*AtCPK3*) may also be a good candidate as it is a stress-responsive gene belonging to the most conserved group of calcium dependent protein kinases (CPKs) [47]. CPKs are ubiquitous genes in plants that directly bind Ca2+ ions before phosphorylating substrates involved in metabolism, osmosis, hormone response, and stress signalling pathways [48,49,50,51,52,53,54]. CPKs have also been reported to be involved in plant defence response [55,56]. Whilst not much is known regarding CPK responses to specific plant virus infections, we have found that *AtCPK3* transcript accumulation changes in response to infection with plant viruses.

In this study, two groups of molecular indicators of virus infection in the model plant *A. thaliana* were examined: the accumulation of LMW RNA using microfluidics technology and the transcript accumulation of virus-response related host plant genes *AtSGS3* and *AtCPK3* using RT-qPCR. The use of these approaches to detect virus infection in *A. thaliana* within broad time-course of infection, from different plant virus infections, was analysed.

## 2. Results

### 2.1. The First Tool: Elevated sRNA Levels Correlate with Virus Infection

#### 2.1.1. Assessment of the Bioanalyzer Method

**Figure 1:**
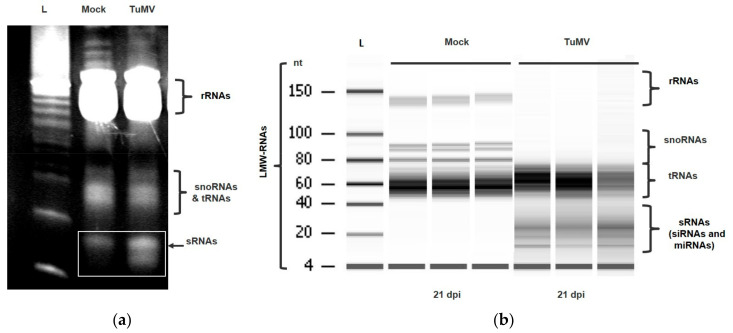
Example of (**a**) agarose gel photo of small RNAs in healthy vs. turnip mosaic virus (TuMV)-infected plant and (**b**) Agilent Bioanalyzer 2100 electrophoretic gel output with low molecular weight (LMW)-RNA components labelled. Three biological replicates of mock and TuMV-inoculated *A. thaliana* LMW-RNA at 21 days post inoculation (dpi). L, Ladder corresponding to 4 to 150 nucleotides (nt), 1, mock-inoculated plant LMW-RNA 1; 2, mock-inoculated plant LMW-RNA 2; 3, mock-inoculated plant LMW-RNA 3; 4, TuMV-inoculated plant LMW-RNA 1; 5, TuMV-inoculated plant LMW-RNA 2; 6, TuMV-inoculated plant LMW-RNA 3. Low molecular weight RNAs (LMW-RNAs), small RNAs (sRNAs; including microRNAs (miRNAs) and small interfering RNAs (siRNAs)), transfer RNAs (tRNAs), small nucleolar RNAs (snoRNAs) and ribosomal RNAs (rRNAs).

Agarose gel electrophoresis comparing healthy and Turnip mosaic virus (TuMV)-infected *A. thaliana* RNA samples suggested an increase in accumulation of sRNAs among infected plants (Figure 1a). Samples were taken at 28 dpi and TuMV infection was confirmed using TuMV specific primers. This finding led to subsequent experiments investigating the use of the Bioanalyzer method to measure the accumulation of LMW-RNA among virus-infected *A. thaliana*.

Figure 1b shows the accumulation of LMW-RNA in three replicate plants that were either mock-inoculated or inoculated with TuMV. The band regions for each component of LMW-RNA (sRNAs, transfer RNAs (tRNAs), small nucleolar RNAs (snoRNAs), and ribosomal RNAs (rRNAs)) were identified as shown in Figure 1b. The electrophoretic gel output suggests differences in the accumulation of all LMW-RNA components when comparing mock-inoculated *A. thaliana* LMW-RNA with that of TuMV-inoculated *A. thaliana*. More specifically, the region of dark banding between 10–40 nt (sRNAs) can be seen clearly in infected plans (Figure 1b lanes 4–6) but is absent from mock-inoculated plants. Conversely, with lanes loaded with equal amounts of LMW RNA, the rRNAs (close to 150 nt) and snoRNAs (70–200 nt) from infected plants appear to be decreased in accumulation compared to the mock-inoculated plants. Moreover, a difference in banding pattern of tRNAs was observed between TuMV-inoculated and mock-inoculated plants.

In order to determine linearity, reproducibility and limits of detection, the mean data of two-fold 10-point serial dilutions of LMW-RNA from three replicate plants of mock-inoculated and TuMV-inoculated 21 dpi were analysed. Supplementary Figure S1a,b present the resultant scatterplots with power trend-lines fitted to illustrate the limits of detection and linearity of the data for the replicate plants. The Bioanalyzer method was able to report concentrations for all 10 dilution points (of the LMW-RNA fraction) for both treatments with consistently high R2 values (R2 minimum 0.979 and maximum 0.996).

In order to determine reproducibility between chips, the mean and standard error of the same biological replicate data of Supplementary Figure S1a,b were calculated and presented in Supplementary Table S1. Analysis of three sRNA chips with three biological replicates of mock-inoculated and TuMV-inoculated LMW-RNA showed little variation between chips at every concentration, as evidenced by minimal error. For the mock-inoculated plants, the minimum error was 0.1 from 234.4 pg of sRNA, while the maximum was 65.2 from 60,000 pg. For the TuMV-infected plants the minimum error was 1.5 from 117.2 pg of sRNA loaded, while the maximum was 508.4 from 30,000 pg. In order to determine between-chip reproducibility, mean and standard error of the mean data of four technical replicates of a two-fold five-point serial dilution of mock-inoculated LMW-RNA 21 dpi were calculated. The data showed negligible variability between chips as evidenced by minimal error (minimum error 20.8 from 1563.0 pg, maximum 299.4 from 6250 pg).

Taken together, the results suggest that the Bioanalyzer method provides data that are linear and reproducible between around 10,000 to 100 pg, with high sensitivity as evidenced by good limits of detection.

#### 2.1.2. Analysis of the Proportion of LMW-RNA Components from Total LMW-RNA

In order to quantify separate LMW-RNA components (sRNA, tRNA, snoRNA and rRNA) from *A. thaliana* total LMW-RNA, samples of three biological replicates of *A. thaliana* plants each inoculated with cauliflower mosaic virus (CaMV), tobacco mosaic virus (TMV), tomato spotted wilt virus (TSWV), turnip mosaic virus (TuMV), turnip yellow mosaic virus (TYMV), or inoculation buffer (mock) were loaded onto sRNA chips for analysis by the Bioanalyzer method. Each LMW-RNA component was analysed as a proportion of total LMW-RNA where the average of all mock-inoculated data at each time point was rescaled to 1.0 (proportion of averaged mock-inoculation). The standard error of the mean was calculated for all virus inoculations at the same dpi.

**Figure 2:**
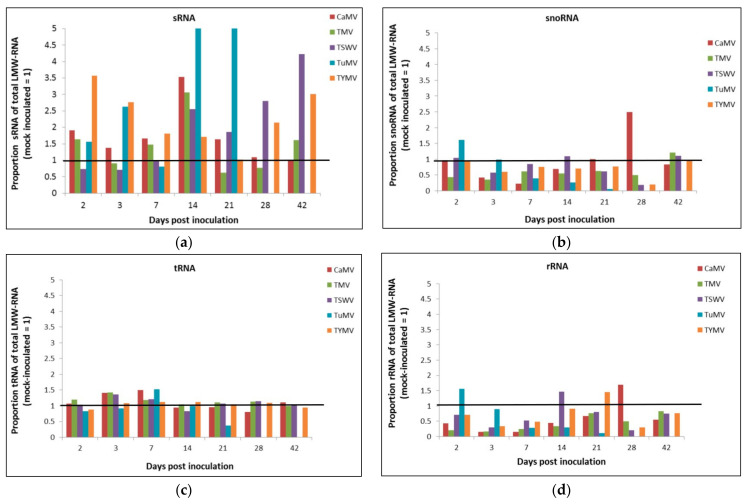
Proportion of low molecular weight (LMW)-RNA components of total LMW-RNA compared to averaged mock-inoculation rescaled to 1.0. (**a**) small RNA (sRNA), (**b**) small nucleolar RNA (snoRNA), (**c**) transfer RNA (tRNA), and (**d**) ribosomal RNA (rRNA). CaMV, cauliflower mosaic virus; TMV, tobacco mosaic virus; TSWV, tomato spotted wilt virus; TuMV, turnip mosaic virus; TYMV, turnip yellow mosaic virus.

Analysing LMW-RNA components as a proportion of averaged mock inoculation enables inference of LMW-RNA component accumulation irrespective of dpi and has the practical advantage of not needing to know the history of the virus infection within a plant. Since the results for the proportion of mock- and virus-inoculation demonstrated the same trend as the results for proportion of averaged mock-inoculation, only the data of each LMW-RNA component as a proportion of averaged mock-inoculated are shown. Figure 2a illustrates the proportion of sRNA in total LMW-RNA compared to averaged mock-inoculation, where an increase is above the line marked at 1 and a decrease is below. All five virus infections resulted in an increase in their proportion of sRNAs but not at every time point; CaMV sRNAs showed an increase in proportion of total LMW-RNA compared to averaged mock inoculation from 2–21 dpi, TMV at 2, 7, 14, and 42 dpi; TSWV between 14 and 42 dpi; TuMV at 2, 3, 14, and 21 dpi; and TYMV at every time point, with the exception of 21 dpi.

Figure 2b illustrates the proportion of snoRNA to total LMW-RNA in virus-infected plants compared to the mock-inoculated plants (averaged). The proportion of snoRNA/LMW-RNA decreased between 3 and 14 dpi in CaMV infected plants; between 2 and 28 dpi in TMV infected plants; at 3, 21 and 28 dpi in TSWV infected plants; between 7 and 21 dpi in TuMV infected plants; and between 3 and 28 dpi in TYMV infected plants. Figure 2c illustrates the proportion of tRNA to total LMW-RNA in virus-infected plants compared to the mock-inoculated plants (averaged). No change was seen in CaMV, TMV, TSWV, and TYMV infected plants while a decrease was observed at 2 and 21 dpi in TuMV infected plants. Figure 2d illustrates the proportion of rRNA to total LMW-RNA compared to the mock-inoculated plants (averaged). A decrease was observed at every time point except 21 dpi in CaMV and TYMV infected plants, at every time point in TMV infected plants, at every time point except 14 dpi in TSWV infected plants, between 3 and 21 dpi in TUMV infected plants.

Taken together, these data suggest a great deal of variability of all components of LMW-RNA at every time point for all virus inoculations. The data for TuMV showed the most consistent profile across the infection time course with the accumulation of sRNAs increasing and the accumulation of tRNA, snoRNA and rRNAs decreasing compared to averaged mock-inoculation, at most time points. It is however notable that the sRNA proportions mostly increased, while rRNA proportions decreased, in response to virus infection.

#### 2.1.3. Ratio of sRNA/rRNA Accumulation as a Tool Predictive of Virus Infection

**Figure 3:**
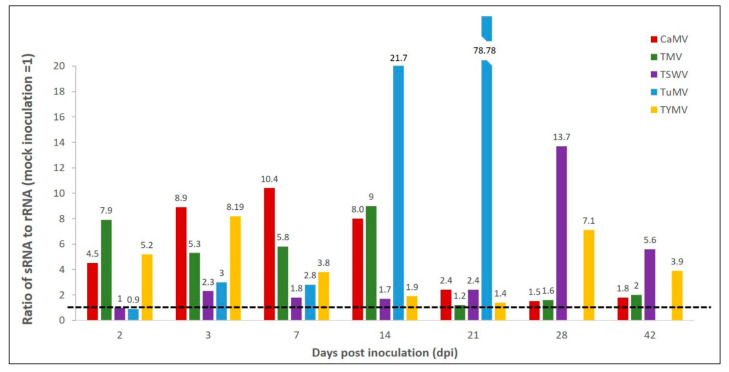
Ratio of sRNA to rRNA of cauliflower mosaic virus (CaMV)-, tobacco mosaic virus (TMV)-, tomato spotted wilt virus (TSWV)-, turnip mosaic virus (TuMV)- and, turnip yellow mosaic virus (TYMV)-inoculated total low molecular weight (LMW)-RNA compared to averaged mock-inoculation across the time course of 2 to 42 days post inoculation (dpi). Specific value given above each bar. Mock-inoculation is rescaled to 1.0. Black line at 1 corresponds to averaged mock-inoculation.

In response to most virus-inoculations, sRNAs appeared to increase in accumulation and rRNAs appear to decrease in accumulation at most time points, compared to averaged mock-inoculation. Hence, the ratio of sRNA to rRNA as a proportion of averaged mock-inoculation was calculated. The aim of this calculation was to determine whether there was a high correlation between a plant being infected and a ratio of sRNA accumulation/rRNA accumulation greater than averaged mock-inoculation rescaled to 1.0, such that this metric could be used as a predictive tool. Figure 3 shows that of 33 samples, 31 were greater than the averaged mock-inoculation, where mock inoculation equals 1.0. This shows that, in this instance, generating a ratio of sRNA to rRNA as a proportion of averaged mock-inoculation correlates with a 94% probability of detecting virus infection across the known virus inoculations and time course of the study. The data for TuMV was quite notable as the ratio of sRNA to rRNA of TuMV-inoculated LMW-RNA compared to averaged mock was 21.69 at 14 dpi and 78.78 at 21 dpi.

### 2.2. The Second Tool: Transcript Accumulation Changes of Virus-Responsive Genes

#### 2.2.1. *AtSGS3* Transcript Accumulation Decreases in Response to Virus Infection

**Figure 4:**
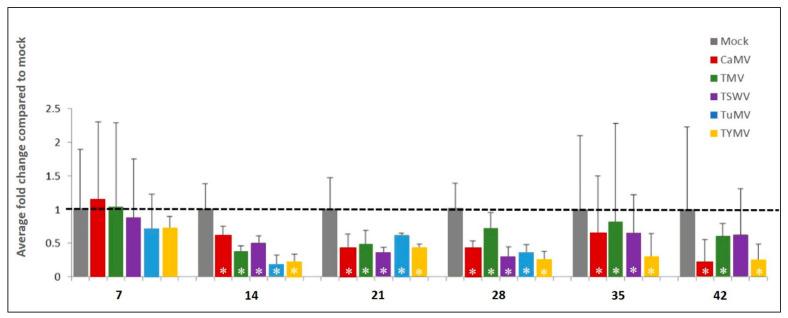
Average fold change of *AtSGS3* transcript accumulation in virus infection. Log transformed, mean centred and auto-scaled *AtSGS3* transcript accumulation of three biological replicates of mock-inoculated and virus-inoculated plants. Data normalised to the reference gene SAND. Virus-inoculated data were compared to mock-inoculated data of the same days post inoculation (dpi) (7, 14, 21, 28, 35 and 42). Error bars are upper 95% confidence level (+95% CI). Asterisks (*) indicate statistically significant difference to the mock in each time point. CaMV, cauliflower mosaic virus; TMV, tobacco mosaic virus; TSWV, tomato spotted wilt virus; TuMV, turnip mosaic virus; TYMV, turnip yellow mosaic virus.

*AtSGS3* transcript accumulation across three replicate plants showed a decreasing trend for transcript accumulation in response to CaMV, TMV, TSWV, TuMV, and TYMV-inoculation at most time points from 14 dpi. While this trend was not of conventional biological significance (arbitrary fold change of ≥2.0), the same trend or biological effect was discernible in the separate biological replicates. Figure 4 presents the fold change in *AtSGS3* mRNA accumulation as average log transformed, mean centred and auto-scaled Q data from the three biological replicates of virus-infected plants compared to mock-inoculated plants of the same time point (dpi). Compared to mock inoculation of the same time point, *AtSGS3* transcript accumulation decreased between 14 and 28 dpi for all viruses except TMV at 28 dpi. The observed fold-change of *AtSGS3* was less than 2.0 in response to CaMV-, TMV-, TSWV-, TuMV-, and TYMV-inoculation compared to mock inoculation of the same time point between 14 and 28 dpi, but these data were statistically significant.

In order to evaluate whether the decrease in transcript accumulation of *AtSGS3* was specific to the biotic stress of virus infection, a further biotic stress of bacterium infection was studied. *Pseudomonas syringae* pv. *tomato* strain DC3000 (*Pst* DC3000) was inoculated to *A. thaliana* plants and compared to mock-inoculated plants. Accumulation of *AtSGS3* mRNA at 1, 2, 6, and 10 dpi was assessed by RT-qPCR. The data presented in Supplementary Figure S2 shows that, compared to mock inoculation, *AtSGS3* accumulation in response to *Pst* DC3000 only increased slightly, with statistical significance only at 2 dpi and not in the other time points.

In order to distinguish biotic and abiotic stress effects on *AtSGS3* transcript accumulation, the abiotic stressors of 100 mM NaCl, 200 mM NaCl, and drought stress were applied. *A. thaliana* plants were either (i) watered following a normal regime (control), or (ii) watered with 100 or 200 mM NaCl (salt stress), or (iii) watering was withheld for 7 d prior to leaf tissue collection at 7, 14, and 21 dpi (drought stress). The data of three biological replicates presented in Supplementary Figure S3 shows that compared to the control treatment there was a statistically significant decrease in *AtSGS3* transcript accumulation at every time point for 100 mM and 200 mM NaCl stress and at every time point for drought stress. The fold change compared to the control treatment in every case was >2.0, showing that these results are of biological significance.

Taken together, these results suggest that *AtSGS3* transcript accumulation consistently decreased to a small degree (<2.0 fold) in response to the five virus infections but to a large degree (>2.0 fold) in response to the abiotic stresses investigated. *AtSGS3* transcript accumulation did not significantly change in response to *Pst* DC3000. Thus, it is possible that a statistically significant small fold change might be indicative of virus infection whereas a large, biologically significant, fold change might be indicative of abiotic stress. However, this possibility needs to be evaluated across further viruses, including cryptic viruses, other plant species, as well as other biotic and abiotic stressors.

#### 2.2.2. *AtCPK3* Transcript Accumulation Increases in Response to Virus Infection

**Figure 5:**
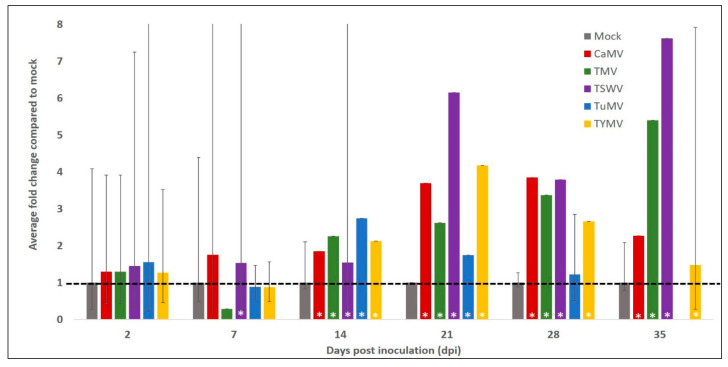
Average fold change of *AtCPK3* transcript accumulation in virus infection. Log transformed, mean centred and auto-scaled *AtCPK3* transcript accumulation of three biological replicates of mock-inoculated and virus-inoculated plants. Data normalised to the reference gene *SAND*. Virus-inoculated data were compared to mock-inoculated data of the same days post inoculation (dpi) (2, 7, 14, 21, 28, and 35). Error bars are upper 95% confidence level (+95% CI). Asterisk (*) indicate statistically significant difference to the mock in each time point. CaMV, cauliflower mosaic virus; TMV, tobacco mosaic virus; TSWV, tomato spotted wilt virus; TuMV, turnip mosaic virus; TYMV, turnip yellow mosaic virus.

To establish the impact on a second transcript following virus infection, the *AtCPK3* transcript was assessed using the same total RNA samples. In response to the same five viruses, *AtCPK3* showed different levels of increase in transcript accumulation in leaves between 14 and 35 dpi (Figure 5). *AtCPK3* appeared to fluctuate within the first week of inoculation, as demonstrated by the high level of variation between the biological replicates. A significant increase in *AtCPK3* accumulation among virus-inoculated plants was seen predominantly at 14 to 28 dpi, although the levels of increase were varied.

Similar to *AtSGS3*, the transcript accumulation of *AtCPK3* in response to other pathogens was also determined (Supplementary Figure S4). Compared to accumulation within mock-treated plants, *AtCPK3* decreased in response to infection with the bacterial pathogen *Pst* DC3000, but the decrease was only significant at 2 dpi. The plants were also challenged with infection with the fungal pathogen *Botrytis cinerea* and compared with mock-inoculated plants. *AtCPK3* mRNA accumulation decreased significantly by about 1.5-fold in response to *B. cinerea* at 2 and 6 dpi and only 0.6-fold by 10 dpi.

The accumulation of *AtCPK3* transcript in response to drought and salt stress was also determined (Supplementary Figure S5a–c and Figure S6a–b. For these experiments, treatments were performed on *A. thaliana* plants grown both in soil and in agar. *AtCPK3* showed small differences in transcript accumulation in leaves in response to salt and drought. Minimal but statistically significant differences were determined at the following time points: about a 1.5-fold decrease at 7 d and 14 d in response to drought (Figure S5a), about 1.3-fold increase at 15 min and about 1.3-fold decrease at 48 h in response to 200 mM mannitol (Figure S6a), and a 1.2- to 1.5-fold increase between 15 min and 4 h in response to 200 mM salt (Figure S6b).

Taken together, these results suggest that AtCPK3 transcript accumulation significantly increased in response to virus at 14 to 35 dpi, and minimally changed in response to Pst DC3000, B. cinerea, drought, and salt stress.

## 3. Discussion

This study investigated the potential use of sRNA and specific mRNA targets as molecular markers that would indirectly indicate virus infection in plants. Analysis of preliminary data obtained from the Bioanalyzer platform with sRNA chips demonstrated that the method had good linearity and good reproducibility both within and between chips and good limits of detection to 0.1 ng. This microfluidics-based method requires only 1 μL of the RNA sample and provides a fast, sensitive and reproducible assay to measure sRNA accumulation. In addition to sRNA quantification, the Bioanalyzer method enables the analysis of all components of LMW-RNA. Data obtained from gene-specific RT-qPCR showed that *AtSGS3* transcript accumulation generally decreased while *AtCPK3* generally increased in *A. thaliana* in response to virus infection.

Analysis of the ratio of sRNA to rRNA to determine correlation between virus infection and sRNA accumulation could be a useful predictive tool for detecting plants infected with virus. The accumulation of sRNAs can increase following virus infection through the processing of viral dsRNA into 21 and 22 nt siRNAs mainly by *DCL4* and *DCL2*, respectively, or their sequestration by virus-encoded suppressors of RNAi according to the type and strength of suppressor encoded by each virus [57,58]. Previously, rRNA has provided a standard reference against which sRNA relative accumulation is established [59]. When calculated, the ratio of sRNA to rRNA as a proportion of averaged mock-inoculation achieved a 94% correlation with known virus infection. Until the ratio of sRNA to rRNA of averaged mock-inoculation is calculated for LMW-RNA within plants subjected to many other biotic and abiotic stresses it cannot be confirmed if this ratio is specific to virus infection. Moreover, while it can be concluded that the Bioanalyzer method can accurately quantify separate LMW-RNA components, not all viruses could be detected at every time point. These findings revealed a limitation in that they are only relative to the mock inoculated plants of the same age and grown under the same conditions. Nevertheless, the analyses of the ratio of sRNA to rRNA as a proportion of averaged mock-inoculation demonstrated that calculating the ratio of these two components correlates with known virus infection among the plants grown and sampled.

The genes investigated in this study, *AtSGS3* and *AtCPK3*, are involved in the RNAi pathway and stress response, respectively. As such, they are good candidates for genes that might be affected by virus infection. Table 1 summarises the transcript accumulation of *AtSGS3* and *AtCPK3* in all treatments. A fold change greater than two was observed in only some virus infections and dpi but the statistical significance among the five virus infections at 14–28 dpi was promising. Moreover, as *AtCPK3* only showed a consistent increase in accumulation in response to virus and decreased or did not change significantly in response to other treatments, this could be a very useful tool for detecting plants with virus infection by comparison to other infections or stresses.

**Figure 6:**
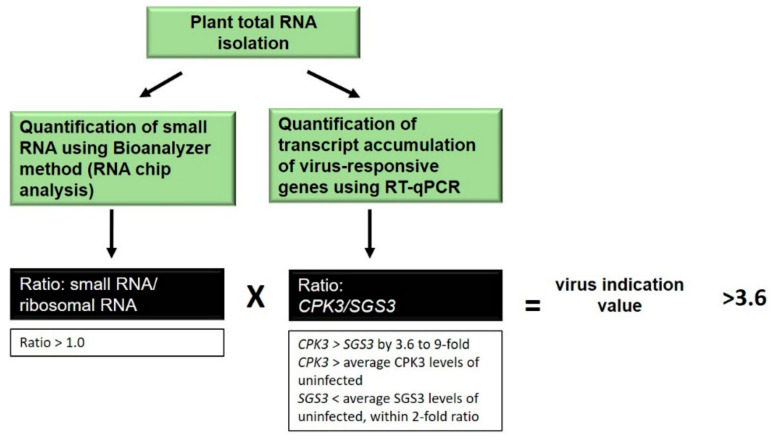
Summary of protocol and example calculation of virus indication value.

From the results of this study, we can propose a methodology and an initial decision tool to determine if plant samples are likely infected by a virus (Figure 6). This is based on the findings that the ratio of sRNA to rRNA of total virus-inoculated LMW-RNA correlated with a 94% probability of detecting known virus infection across the present inoculations and time course; that the *AtSGS3* transcript decreased between 14 and 28 dpi for all viruses; and that *AtCPK3* transcript increased between 14 and 28 dpi for all viruses. Following total RNA isolation, aliquots of the samples can then be analysed using Bioanalyzer to determine the amount of LMW-RNA, as well as using Rt-qPCR measuring virus-responsive genes such as *AtSGS3* and *AtCPK3*. A ratio of >1.0 for sRNA/rRNA is correlated to virus infection, this ratio can be one of the factors that can be used in the equation. A potential calculation can consist of multiplying this with the ratio of *AtCPK3*/*AtSGS3*. Since *AtCPK3* increased between 1.6 to 7-fold in response to virus and *AtSGS3* decreased by less than 2-fold, the minimal indication value after multiplying these ratios calculated as 3.6. This may then be used as a cut-off value to determine if a plant is infected with a virus. Values above this number will indicate that the plant is highly likely to be infected. This value will of course change based on the data that will be available from future research looking at these and other potential molecular indicators of virus infection. Moreover, other calculation methods such as probability-based calculations may also be investigated in the future.

The research was undertaken with five unrelated viruses from diverse families that include both RNA (positive sense and ambisense) as well as DNA genomes. Therefore, the molecular responses that are common across these infections and across time reveal basic interactions between plant viruses and their host plant. Moreover, further study examining multiple infections as well as latent infections are necessary as these may trigger different gene expression profiles. This method may prove to be a useful tool to link pathogenicity with presence of viruses and thereby complement the information gained by high throughput sequencing, which is typically bereft of biological significance of virus presence.

This study used only a single plant host to determine whether a broad range of plant pathogenic viruses could be detected using non-specific tests for virus infection. Further refinement to confirm that the method is widely effective includes testing the proposed method on other plant species whose genomes have been sequenced and represent major taxonomic groups and developing degenerate primers for *AtSGS3* and *AtCPK3* homologues that are sensitive and specific for a wide range of plant species. Moreover, as the study was only conducted on glasshouse grown test plants under controlled conditions, this method still needs to be tested on field collected samples or border security samples with infections of unknown age and from varied environments. Further studies comparing the accumulation of these host molecular indicators before and after symptom appearance, as well as comparing symptomatic and asymptomatic or latent infections will also be important.

Other molecular markers and approaches may also be explored, considering recent studies that have been published after we have carried out the experimental work for this study. Other genes have been reported to be involved *SGS3*/*RDR6* pathways and can be investigated for differential expression in response to other plant virus infections. A calmodulin-like protein from *N. benthamiana* acts a suppressor of RNA silencing by degrading *SGS3* during infection with a geminivirus; hence may be elevated in response to many other virus infections [60]. A virus-induced small peptide 1 (VISP1) that inhibits siRNA amplification by degrading *SGS3*/*RDR6* bodies may also be a potential host marker of virus infection [45]. On the other hand, plant receptor for activated C kinase 1 (*RACK1*) has also recently been reported to be downregulated by virus-induced gene silencing, and acts a s a bridge between a viral replication protein and a CPK [55]. A reduction in ROS accumulation may also be explored as an indicator of virus infection as the production of these molecules have been reported to be inhibited by some viruses and induced by bacterial pathogens [61]. A microtitre-based plate assay to screen a large collection of candidate host pattern-recognition receptors (PRR) and measure pattern-triggered immunity (PTI) activation has been developed recently [62]. This assay measured the activity of plant peroxidase (*POX*) produced in response to flagellin 22 (flg22) and has been optimised to minimise the amount of plant tissue required. It would be valuable to test this approach to viral infections. Physiological and anatomical parameters that change as part of the plant immune defence system against virus infection should also be considered for future research [63,64,65,66,67].

Although there are many limitations and factors yet to investigate, this study is the first to put emphasis on the potential use of molecular markers for detecting generic virus infection by monitoring the host plant rather than the virus. Such a generic molecular response to virus infection may be eventually used for in-field, remote testing using technologies such as aptamers or nanoprobes linked with nucleic acid probes [68,69]. The use of these genetic markers in conjunction with these advances in technology may provide a very rapid diagnostic tool for plant virus infections in the field or in border areas.

## 4. Materials and Methods

### 4.1. Plant Material

*A. thaliana* (col-0) plants were infected separately with tobacco mosaic virus (TMV), cauliflower mosaic virus (CaMV), tomato spotted wilt virus (TSWV), turnip mosaic virus (TuMV), and turnip yellow mosaic virus (TYMV). Leaf samples were taken at 2-, 3-, 7-, 14-, 21-, 28-, 35-, and 42-days post inoculation (dpi) with three biological replicates of virus- and mock-inoculated plants for each time point. Drought experiments, fungal infection with *Botrytis cinerea*, and bacterial infection with *Pseudomonas syringae* pv. *tomato* strain DC3000 were also performed to determine plant responses that are unique to virus infection and not characteristic of other stress and pathogen responses. For drought, samples were taken at 7, 14, and 21 d because most plants appeared desiccated after 21 d. For fungal and bacterial infections, samples were taken at 1, 2, 6, and 10 dpi as most plant tissue had been destroyed by the infection by 10 dpi. Three biological replicates were sampled for treated and untreated plants at each time point.

### 4.2. sRNA Isolation

LMW-RNA was isolated from *A. thaliana* leaf tissue samples using the mirPremier™ microRNA isolation kit (Sigma-Aldrich, MO, USA). This kit provided the most consistent yields of sRNA and best quality compared with two other kits, as determined by NanoDrop spectrophotometry (ND-1000 Spectrophotometer; Nanodrop Technologies Inc., Wilmington, DE, USA). Procedures were completed according to the manufacturer’s protocol. All samples had an absorbance ratio (A260:A280) of between 1.8 and 2.2 where a ratio of ~2.0 is generally accepted as ‘pure’ or of good quality for RNA. Following quantification, all LMW-RNA samples were normalised to 30 ng/μL.

### 4.3. Total RNA Extraction, Quality Analysis and cDNA Synthesis

Total RNA was extracted from the leaf or root samples collected as described above using a Spectrum TM Plant Total RNA Kit (Sigma-Aldrich, St. Louis, MO, USA) following the manufacturer’s instructions. RNA samples were treated with DNase I (amplification grade; Invitrogen, San Diego, CA, USA) to remove any potential genomic DNA (gDNA) contamination. RNA concentration and purity were measured using a Nanodrop ND-1000 spectrophotometer (Nanodrop Technologies Inc., Wilmington, DE, USA) while RNA integrity was analysed using a Bioanalyzer 2100 RNA Nano LabChip 6000 (Agilent Technologies, Santa Clara, CA, USA). RNA Integrity (RIN) values were assigned by the Bioanalyzer software algorithm, which determines the quality of a total RNA sample from the 28S:18S ribosomal RNAs ratio and from the entire electrophoretic profile (Schroeder et al. 2006). RIN values range from 1 to 10, with 10 being a fully intact RNA and ≥7.0 being acceptable. However, the algorithm used by the software version was designed for mammalian RNA and did not consider chloroplast RNA in plants. RIN values were then adjusted with visual inspection of the peaks for 25S, 23S, and 16S RNAs. All RNA samples used for the succeeding experiments had an absorbance ratio (A260:A280) between 1.8 and 2.2, and adjusted RIN value of 7.0 or greater.

RNA samples were reverse transcribed into cDNA using a SuperScript^®^ VILO™ cDNA synthesis kit (Life Technologies-Invitrogen, San Diego, CA, USA) following the manufacturer’s protocol. The total amount of RNA transcribed to cDNA was adjusted to 2 µg, in a total volume of 40 µL.

### 4.4. Small RNA Lab on Chip Protocol

Small RNA chips were loaded following the manufacturer’s Small RNA Kit Quick Start Guide. The chip was run in the Bioanalyzer instrument with the appropriate assay, sRNA, selected from the software menu. A successful sRNA run results in one marker peak and two regions that are defined arbitrarily by the Bioanalyzer expert B02.04 software: the LMW-RNA region from 0 to 150 nucleotides (nt), and the microRNA (sRNA) region from 10 to 40 nt. These regions were modified by selecting the region table within the software and setting regions for analysis to extend the LMW-RNA region to 200 nt and to encompass the composition of LMW-RNA components; that is 50–80 nt for transfer RNAs (tRNAs of 73–94 nt), 85–100 nt for small nucleolar RNAs (snoRNAs, e.g., U14 of 84–106 nt) and 110–180 nt for ribosomal RNAs (rRNAs; 5.8 S of 154 nt and 5 S of 120 nt) in addition to the sRNA region already set (10–40 nt). The LMW-RNA component regions were set to encompass the average nt size reported for each LMW-RNA component across the literature [70,71].

Regression analysis was performed to estimate the relationship between each LMW-RNA component (sRNA, tRNA, snoRNA, and rRNA) and each virus-inoculated and mock-inoculated LMW-RNA from 120 observations. Mixed model repeated measures analyses of fixed (LMW-RNA components) and random (three biological replicates) effects was kindly performed by Dr Nihal de Silva using the statistical analysis software system SAS/STAT 9.22 to determine if there was a difference in LMW-RNA components profile between each of the five virus-inoculations and mock-inoculation across the 2 to 42 dpi time course. To establish if the measured load on the two components differed, principal component analysis was performed on the entire data set, thus converting the complex and multidimensional data into a lower number of variables. If the load between the two calculated components differed it could be determined that the accumulation of LMW-RNA components of the five virus-inoculations can be distinguished from mock-inoculation.

### 4.5. RT-qPCR

Gene specific qPCR primers were designed specifically near the 3′ end for *AtSGS3* (F2352: GGAGAATTTCGAGATGTTGCAG; R2599: CCACAAACTCCTCCATCTCT TT) and *AtCPK3* (F1526:5′CATTGCTGAAGTAGACACCG3′; R1640:5′GATCTCTCA CATTCTGCGTC3′). Primers were initially tested for their ability to amplify a product of correct size by employing an end-point RT-PCR reaction consisting of 1.0 μL cDNA synthesised from 2 µg RNA, 12.5 μL of GoTaq^®^Green Master Mix (Promega Corp., Madison, WI, USA), 0.5 µL of each 10 µM stock forward and reverse primer, and 10.5 µL of UltraPure™ DNase/RNase-free distilled water (Life Technologies—Invitrogen San Diego, CA, USA) to a total volume of 25 µL. The following PCR conditions were employed: initial denaturation at 94 °C for 5 min, followed by amplification with 35 cycles of denaturation at 94 °C for 30 s, annealing at 60 °C for 30 s and extension at 72 °C for 30 s, followed by a final extension at 72 °C for 5 min. Negative control reactions, omitting cDNA template, were also prepared for each set of reactions.

To quantify the transcript accumulation of the target and reference genes from each sample, qPCR reactions were performed using a LightCycler 480 Real-Time PCR system (Roche Applied Science, Branchburg, NJ, USA). Reactions were in a 10 μL total volume containing 1 μL of primer pair (2 μM stock forward and reverse primer), 4 μL of cDNA, and 5 μL of LightCycler 480 SYBR Green I Master mix reagent (Roche). A Biomek 3000 Robot (Beckman Coulter, Fullerton, CA, USA) was used to aliquot all reagents, primers, and samples into 384-well plates, with two technical replicates and three biological replicates for each sampling time point. The qPCR reaction consisted of pre-incubation at 95 °C for 5 min and amplification with 45 cycles of denaturing at 95 °C for 10 s, annealing at 60 °C for 10 s, and extension at 72 °C for 10 s. Fluorescence acquisition was set up at the end of each cycle. The amplification step was followed by a melting curve analysis, with one cycle of 95 °C for 5 s, 65 °C for 1 min, and a ramp to 97 °C at a rate of 0.11 °C/s. Five fluorescence acquisitions per °C were taken. Samples were cooled at 40° for 10s.

Fluorescence data per cycle were exported from the LightCycler 480 software into a *.csv file using Python 2.6.3 (Python Software Foundation; custom script by Jeremy McRae, The New Zealand Institute for Plant and Food Research). Transcript accumulation of the targeted genes were normalised using the reference genes F-BOX family protein (FBOX), SAND family protein (SAND), and elongation factor 1 alpha (EF1 α). These have been previously validated as good reference genes for virus infection in *A. thaliana* [72]. Baseline correction, log transformation and primer PCR efficiency calculation from linear regression were done using the software LinRegPCR 11.1. Initial expression values or transcript abundance (Q) for each gene for each sample were calculated using the formula shown in Supplementary Figure S7. This calculation employs the comparative ΔCq method and rescaling of the data based on the calculated PCR efficiency and the relative accumulation values for each gene. The M value for each reference gene and normalisation factor for each Q value was calculated using GeNorm v3.5 analysis software. Reference genes with an M value less than 1 were considered as acceptable for use in the normalisation of qPCR data. Data sets were then standardised using a series of sequential corrections including log transformation, mean-cantering, and autoscaling as described by Willems et al. [73]. Figures were generated from the analysis using the most stable reference gene (lowest M value). Statistical support to results was determined using Analysis of Variance (ANOVA) and follow-up tests such as Tukey’s test and Fisher’s LSD. Statistical support was considered as strong (*P* ≤ 0.01), good (0.01< *P* ≤ 0.05) or weak (0.05 < *P* < ~0.10). Levene’s test was initially done before the ANOVA test to determine if the values have equal variance. The statistical software Minitab was used to perform all statistical tests (Minitab 17 Statistical Software 2010).

## Figures and Tables

**Table 1:** Transcript accumulation of *AtSGS3* and *AtCPK3* in response to viral, bacterial, fungal and abiotic stresses. Viruses tested were cauliflower mosaic virus (CaMV), tobacco mosaic virus (TMV), tomato spotted wilt virus (TSWV), turnip mosaic virus (TuMV) and turnip yellow mosaic virus (TYMV).

Treatments	*AtSGS3* Transcript Accumulation	*AtCPK3* Transcript Accumulation
Viruses	Decrease by less than 2.0-fold	Increase by about 1.6 to 7-fold
*Pseudomonas syringae* DC3000	Increase by less than 2-fold, or no change	Decrease by about 1.9-fold
*Botrytis cinerea*	Not tested	Decrease by about 2.9-fold
Drought	Decrease by greater than 2-fold	Decrease by about 1.5-1.7-fold
Salt	Decrease by greater than 2-fold	Decrease by less than 2-fold

## Data Availability

The data is contained within this article.

## Supplementary Materials

The following are available online at https://www.mdpi.com/article/10.3390/plants11020188/s1, Figure S1: Scatterplots of 10-point two-fold serial dilutions of mock- and Turnip mosaic virus (TuMV)-inoculated A. thaliana low molecular weight (LMW)-RNA at 21 dpi, Table S1: Amount of small RNA loaded, amount reported (pg) and standard error of the mean of replicates of low molecular weight (LMW)-RNA, Figure S2: Normalised average Q of AtSGS3 transcript accumulation in response to Pseudomonas syringae pv. tomato strain DC3000 (Pst DC3000) compared to mock-inoculation, Figure S3: Normalised average Q of AtSGS3 transcript accumulation in response to NaCl and drought stress, Figure S4: AtCPK3 transcript accumulation in A. thaliana leaves in response to Botrytis cinerea and Pst DC3000, Figure S5: AtCPK3 transcript accumulation in A. thaliana leaves in response to drought and high salt, Figure S6: AtCPK3 transcript accumulation in A. thaliana leaves in response to 200 mM mannitol and 200 mM salt, Figure S7: Formula used to normalise transcript abundance data (Q).
