# Supplementary material for: The Potential of Molecular Indicators of Plant Virus Infection: Are Plants Able to Tell Us They Are Infected?
Source: Plants (Basel). 2022 Jan 11;11(2):188. doi: 10.3390/plants11020188 (PMC8777591; doi:10.3390/plants11020188)
Supplement: Supplementary file 1 [file plants-11-00188-s001.zip › Supplementary files/Supplementary Figures & Tables.pptx]

## Slide 1
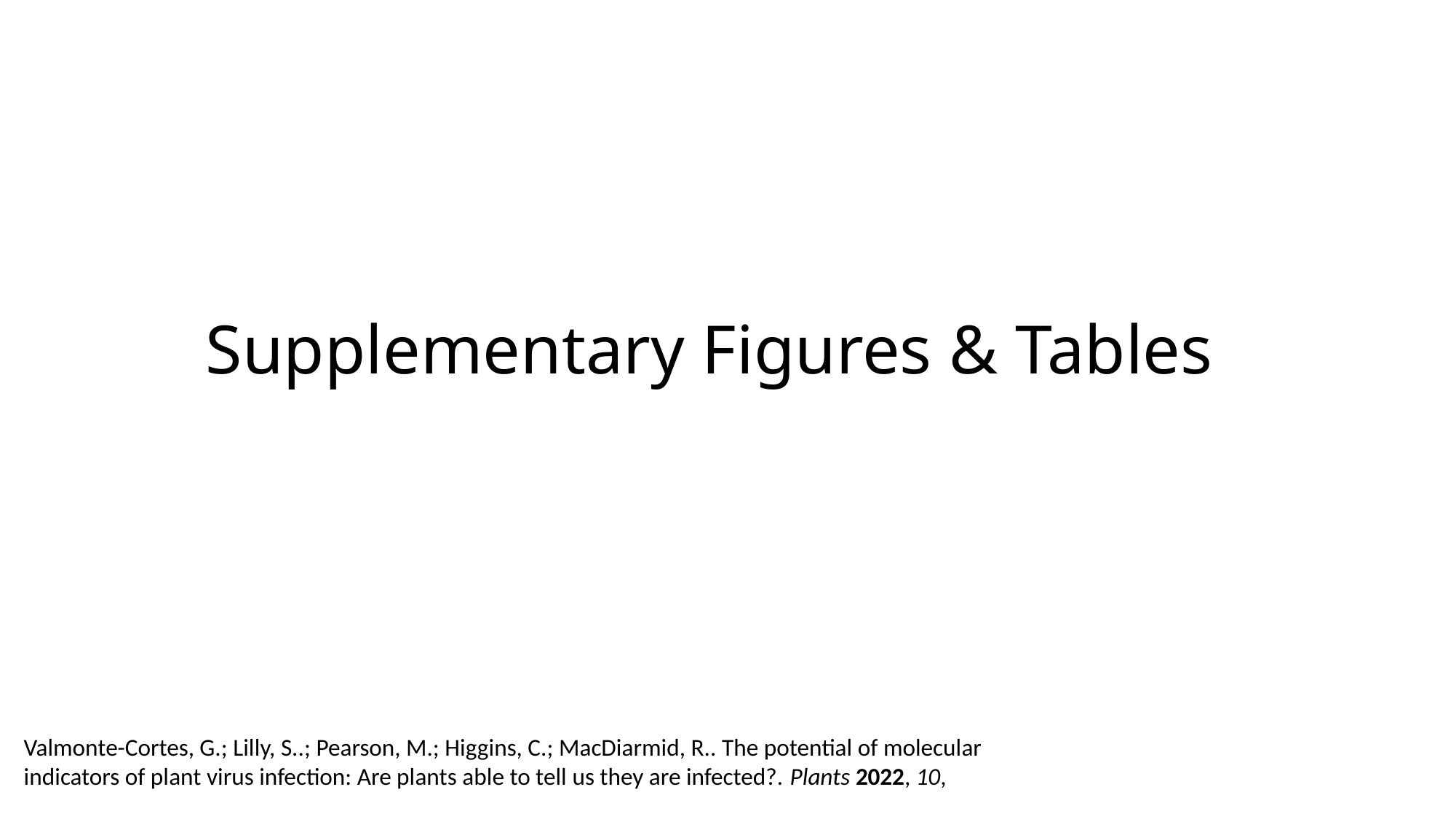

# Supplementary Figures & Tables
Valmonte-Cortes, G.; Lilly, S..; Pearson, M.; Higgins, C.; MacDiarmid, R.. The potential of molecular indicators of plant virus infection: Are plants able to tell us they are infected?. Plants 2022, 10,

## Slide 2
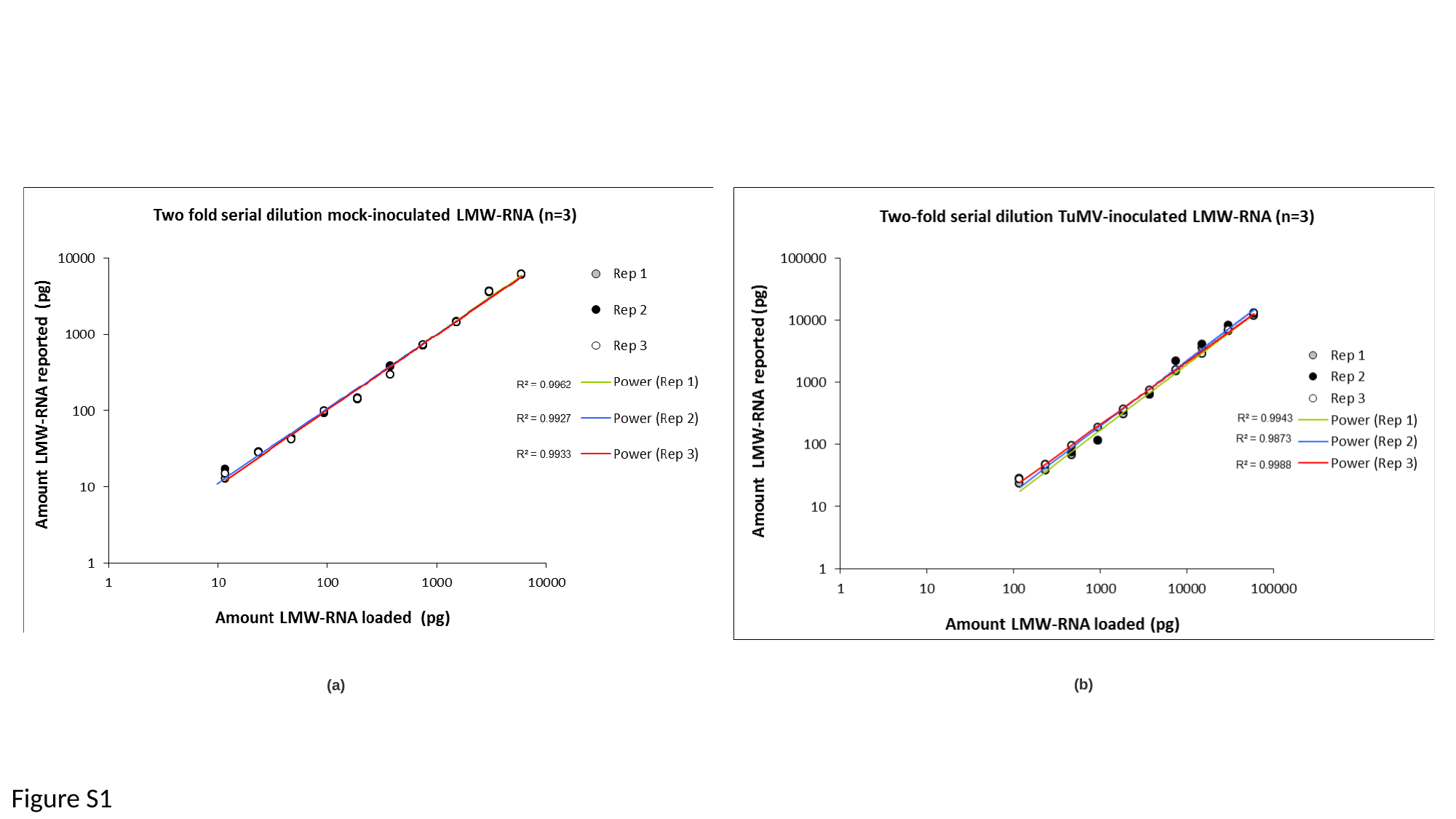

(b)
(a)
Figure S1

## Slide 3
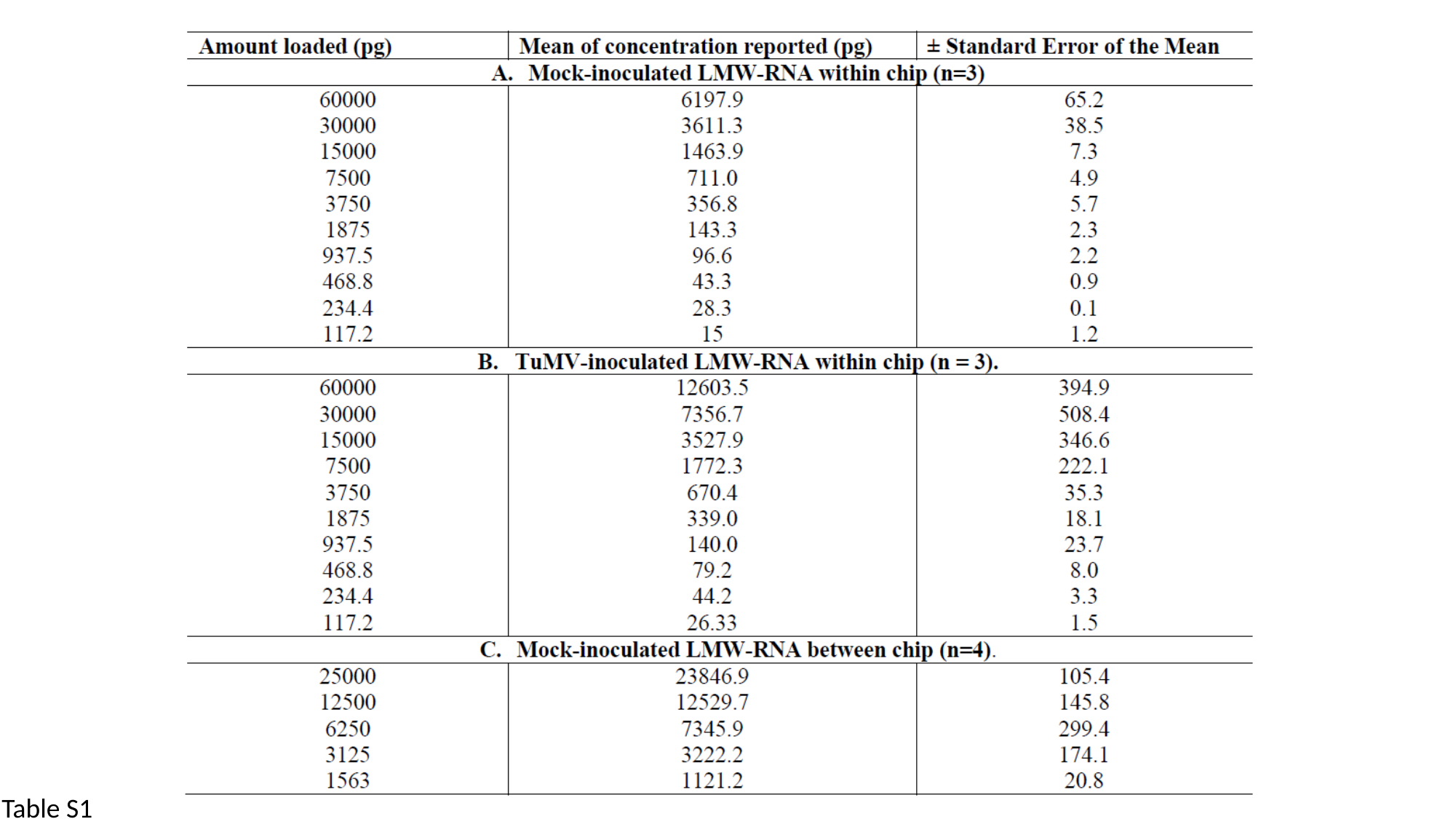

Table S1

## Slide 4
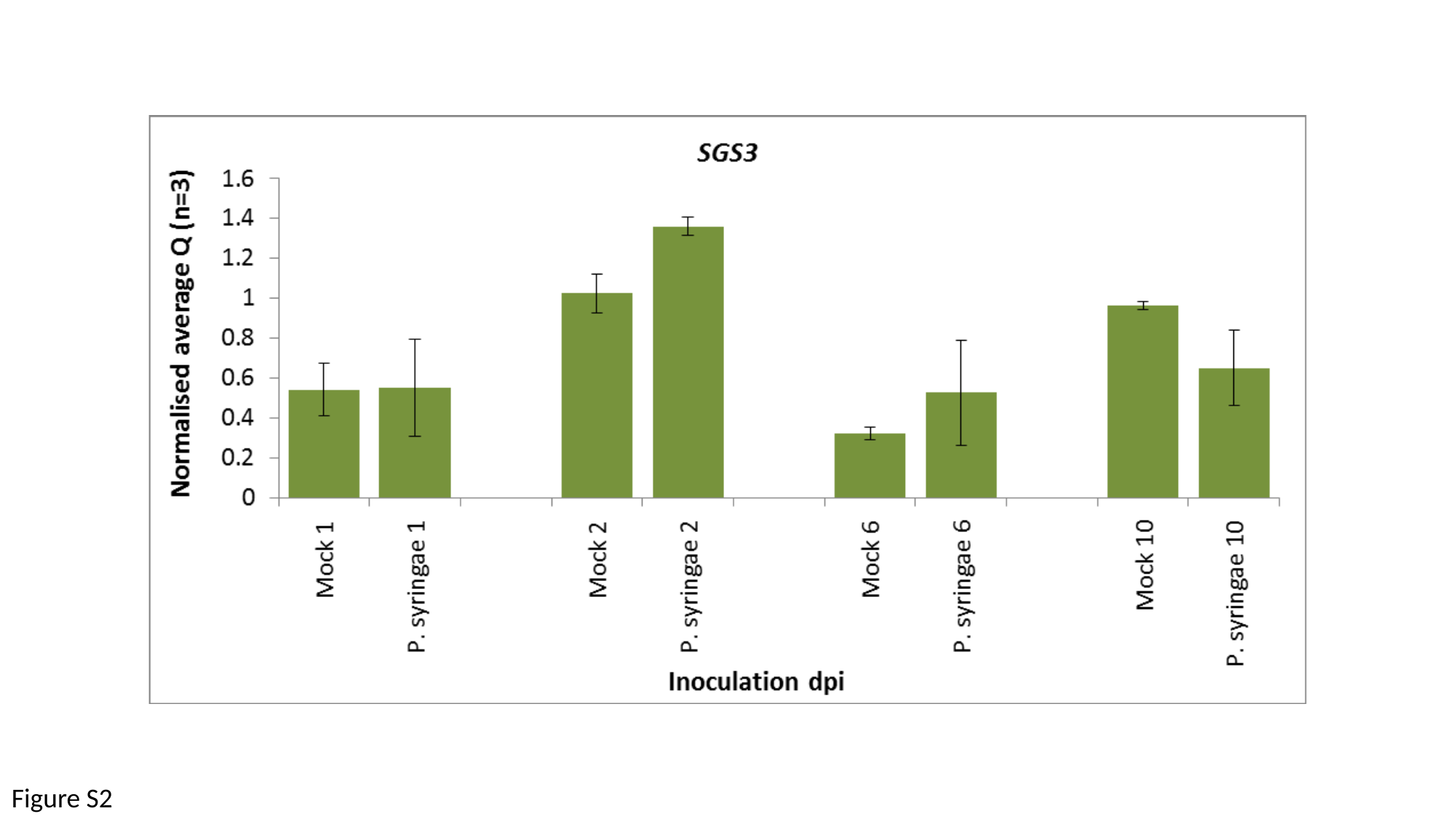

Figure S2

## Slide 5
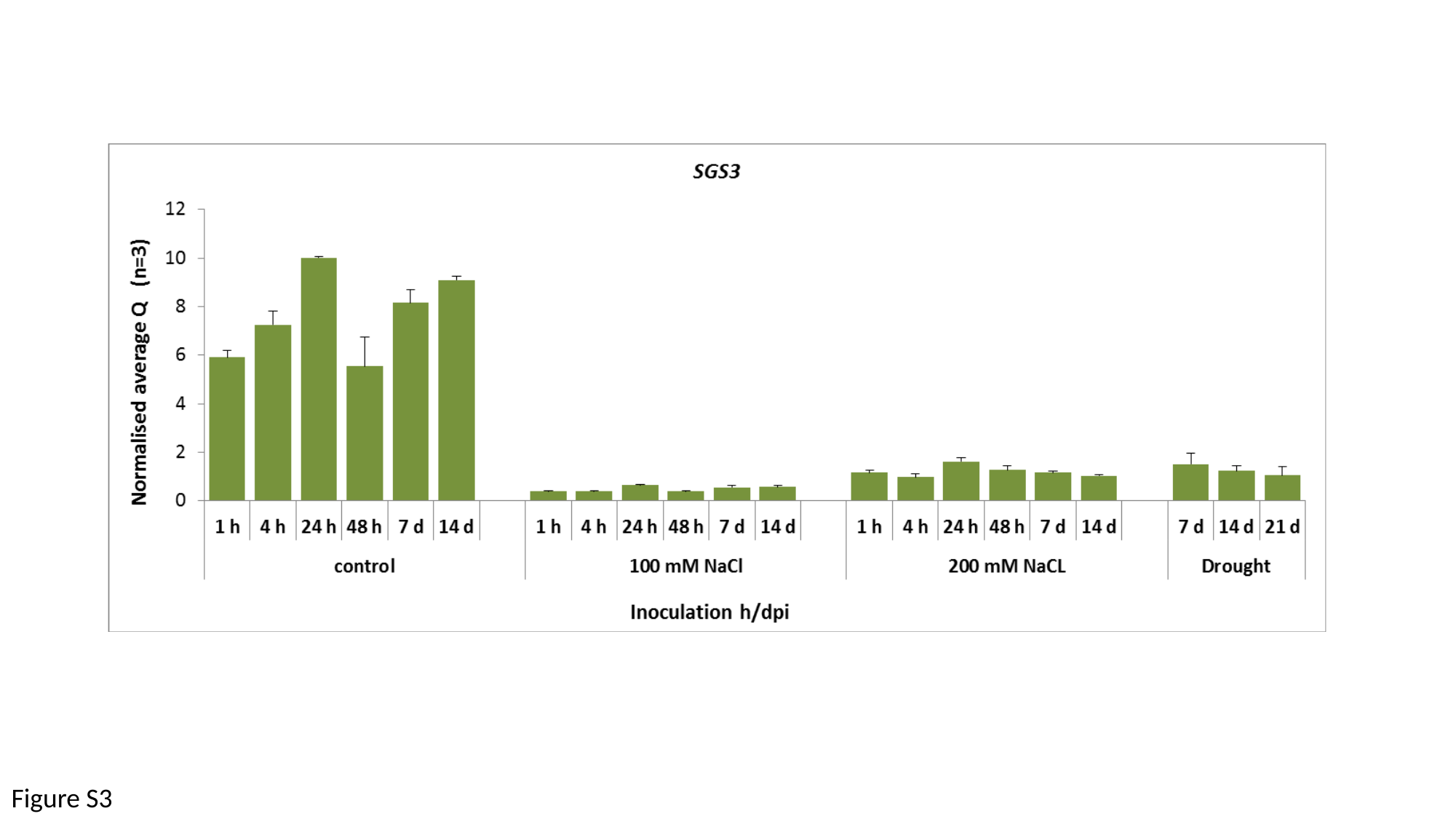

Figure S3

## Slide 6
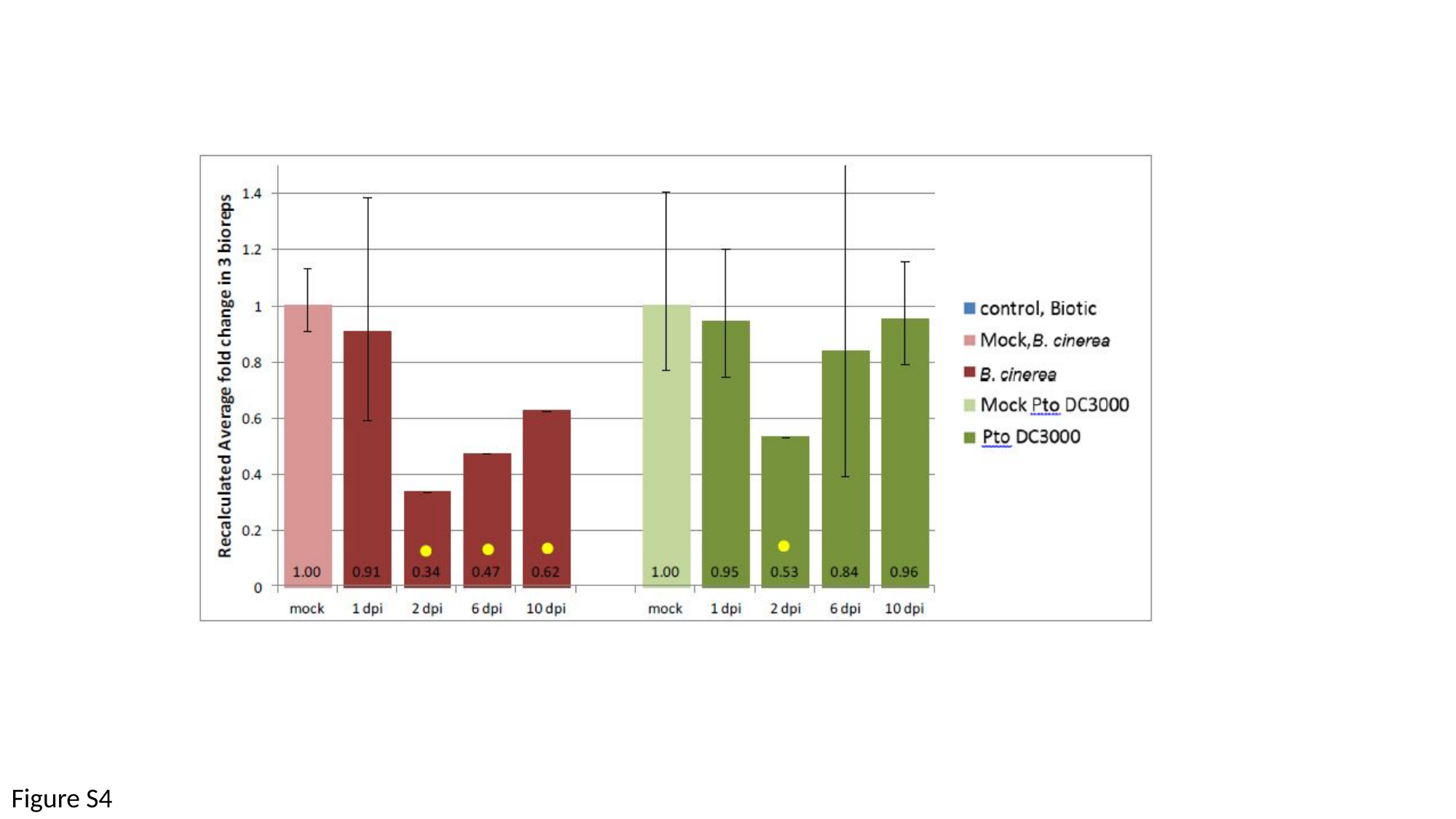

Figure S4

## Slide 7
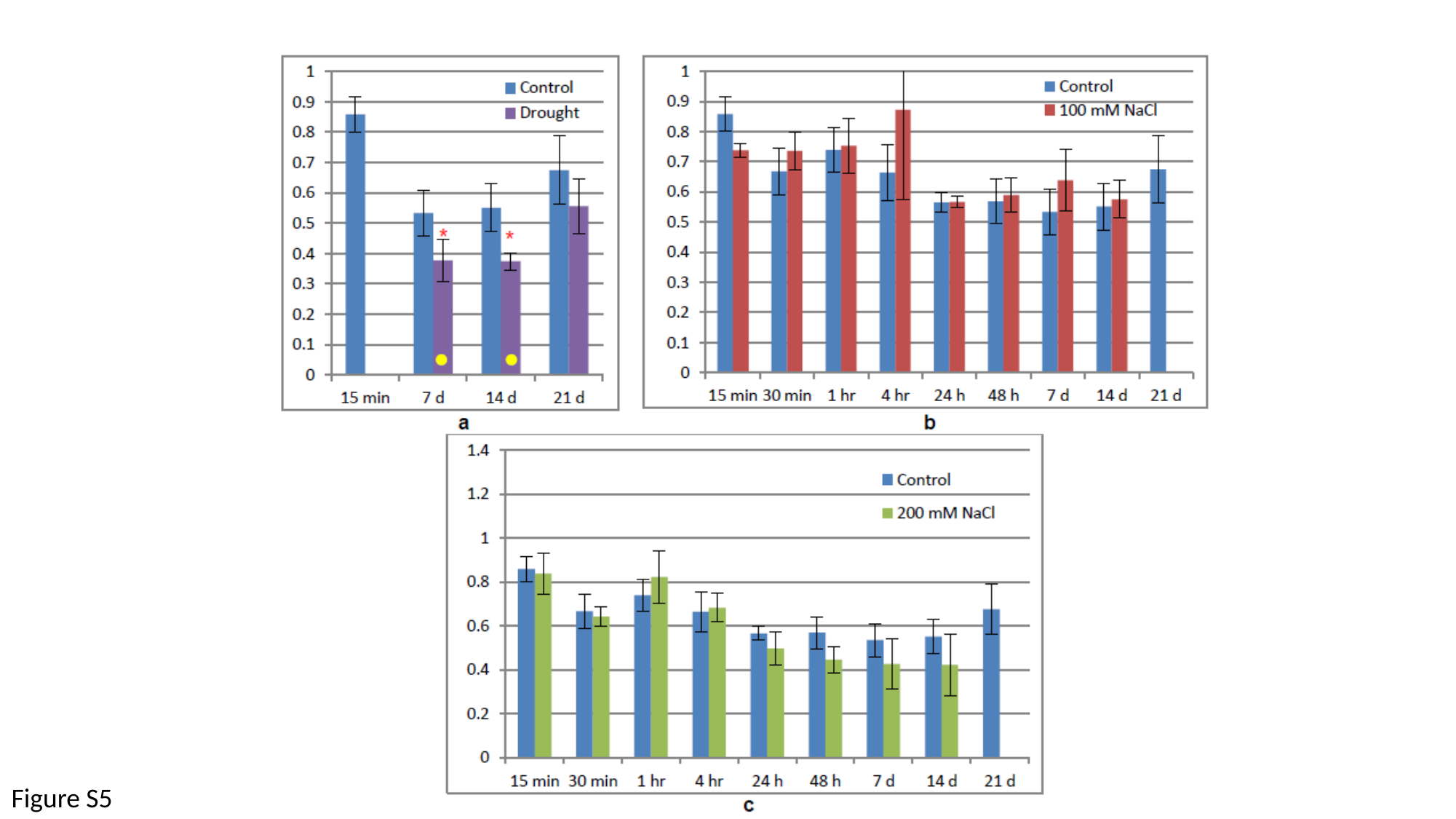

Figure S5

## Slide 8
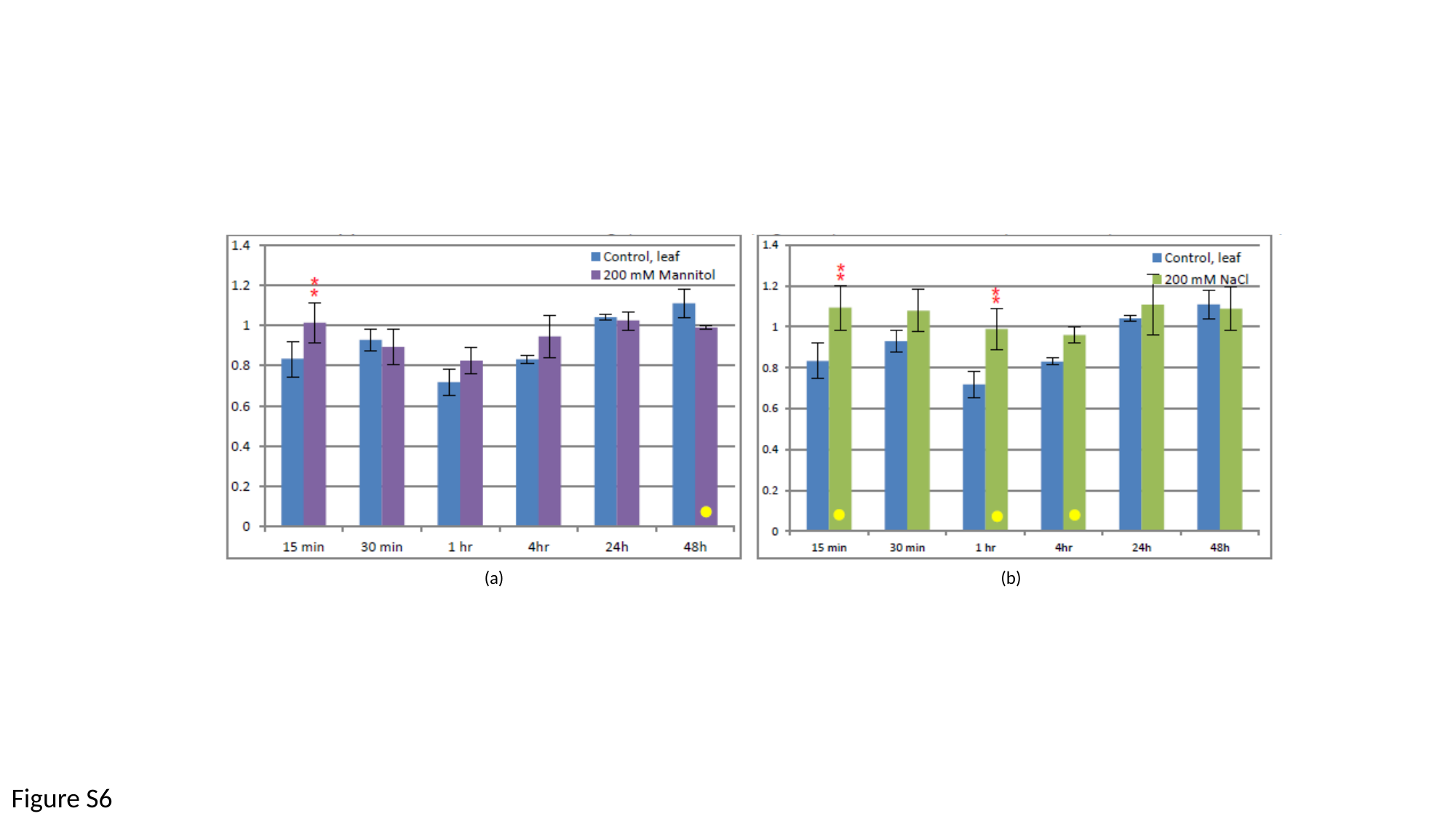

(b)
(a)
Figure S6

## Slide 9
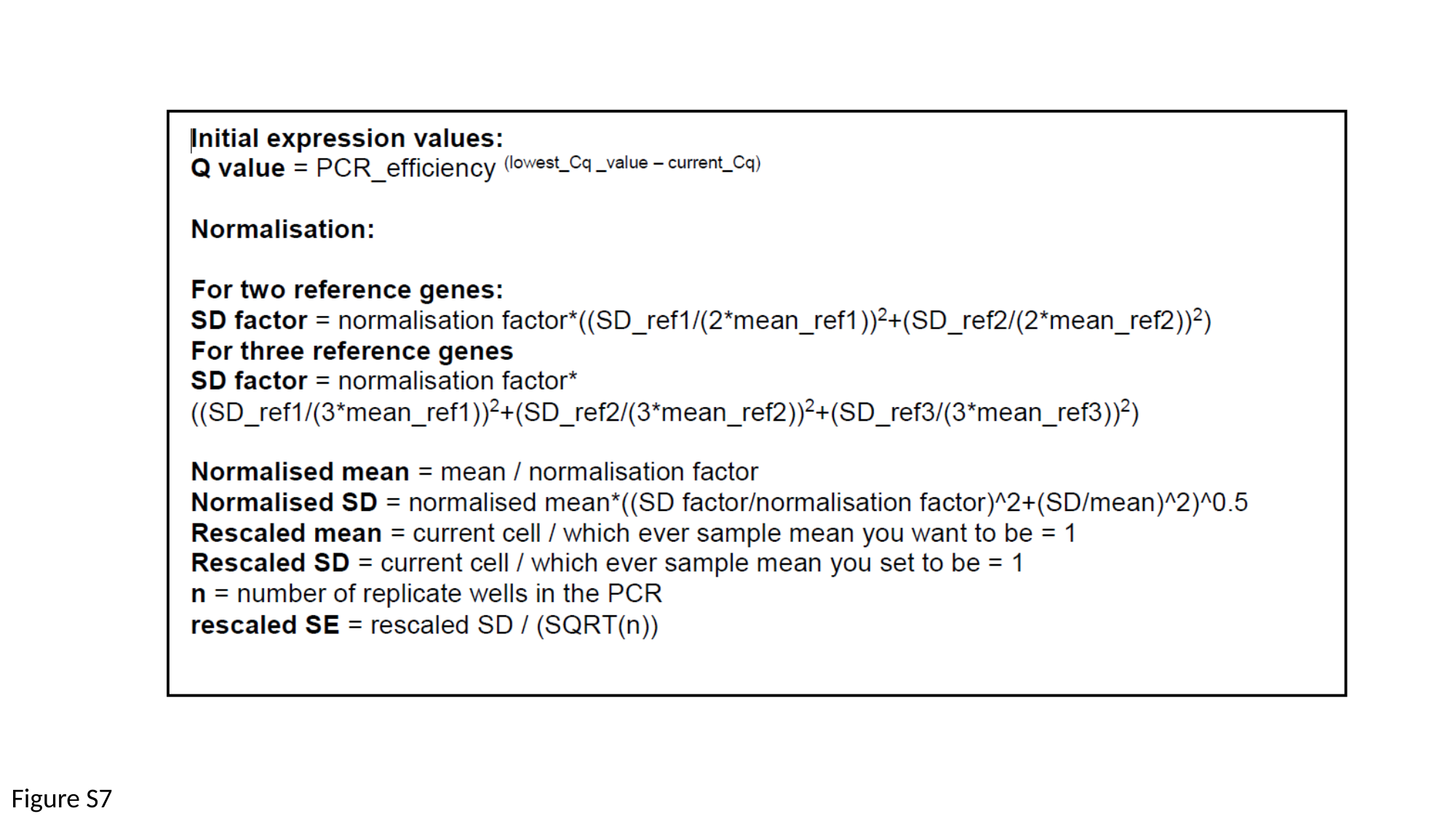

Figure S7
